# A case of chronic inflammatory demyelinating polyneuropathy with reversible alternating diaphragmatic paralysis: case study

**DOI:** 10.1186/s13089-015-0033-5

**Published:** 2015-10-21

**Authors:** Kavi Haji, Ernest Butler, Colin Royse

**Affiliations:** Intensive Care Unit, Frankston Hospital, Peninsula Health, Hastings Road, Frankston, VIC 3199 Australia; Department of Neurology, Frankston Hospital, Peninsula Health, Frankston, VIC Australia; Department of Surgery, The University of Melbourne, Melbourne, VIC Australia; Department of Anaesthesia and Pain Management, The Royal Melbourne Hospital, Melbourne, VIC Australia

**Keywords:** CIDP, Diaphragm, Phrenic nerve, Ultrasound, Mechanical ventilation

## Abstract

Respiratory failure requiring mechanical ventilation has been reported in patients with bilateral diaphragmatic paralysis due to CIDP. We report a case of CIDP that progressed to respiratory failure with normal chest radiography despite unilateral diaphragmatic paralysis. This manifestation would have been missed if ultrasound was not employed.

**Electronic supplementary material:**

The online version of this article (doi:10.1186/s13089-015-0033-5) contains supplementary material, which is available to authorized users.

## Background

Diaphragmatic paralysis can result from neurological diseases affecting the phrenic nerve including Guillain–Barré syndrome, and it has also been described in chronic inflammatory demyelinating polyneuropathy (CIDP) [1, 2]. Although rare, there have been reports of acute respiratory failure requiring mechanical ventilation with bilateral phrenic nerve involvement in CIDP [3]. We report a case of CIDP that manifested an alternating hemidiaphragmatic paralysis. The patient developed acute respiratory failure requiring mechanical ventilation and a prolonged intensive care unit (ICU) stay. The person responsible for the patient and the patient after discharge from ICU gave consent for the authors to publish the report.

## Case presentation

A 42-year-old male presented to the emergency department following acute onset of breathlessness. He had a history of unexplained lower limb paraplegia at 29 years of age. His other past medical history included ischaemic heart disease and diet-controlled diabetes mellitus. He was an active smoker. Over 4 months prior to admission, the patient had multiple presentations with variable non-specific relapsing complaints including progressive upper limbs weakness, transient left eye diplopia, vague complaints of lethargy, musculoskeletal pain, and alternating urinary retention and incontinence.

During this admission, the patient developed progressive severe upper limb weakness, areflexia and altered sensation. He was dysphonic and he had a weak cough and a weak palatal reflex. The patient developed hypercarbic respiratory failure and was admitted to ICU where he received non-invasive ventilation. His serial arterial blood gas analyses and other clinical data are shown in Table 1. A few hours after admission to ICU the patient became progressively obtunded which resulted in altered consciousness and he was subsequently intubated. His chest X-ray was unremarkable and blood sugar level was 5.9 mmol/L. The patient was mechanically ventilated with synchronized intermittent mandatory ventilation mode (SIMV) with pressure support ventilation (PSV).

**Table 1:** Patient’s data

Data	Admission day	Day 1	Day 2	Day 10
RASS	−3	−2	−2	0
Sedation	None	Propofol	Midazolam and morphine	Midazolam and morphine
Ventilation mode	15 L/min of O_2_ via hudson mask	SIMV volume control	PSV	PSV
Tidal volume (mL)	Not ventilated	500	590	467
Respiratory rate (breath/minute)	30	16	19	23
PS (cmH_2_O)	0	12	15	10
PEEP (cmH_2_O)	0	10	5	5
FiO_2_ (%)	0.6	0.4	0.3	0.3
pH	7.19	7.46	7.37	7.43
PaO_2_ (mmHg)	190	96.7	73.7	107
PaCO_2_ (mmHg)	73	37.4	52	35
HCO_3_ (mmHg)	27	26.9	27	25
Left hemidiaphragm (cm)	Not studied	0	1.8	0.6
Right hemidiaphragm (cm)	Not studied	1.9	0	0.8

Normal value for hemidiaphragm excursion is 0.9–2 cm (3, 8)

*RASS* Richmond analgesia and sedation scores, *SIMV* synchronized intermittent mandatory ventilation, *PSV* pressure support ventilation, *PS* pressure support ventilation

Within 24 h, the patient’s gas exchange improved dramatically and ventilation mode was quickly weaned from SIMV to PSV mode only. He was receiving fractional inspired oxygen 30 %, positive end-expiratory pressure of 5 cmH_2_O and pressure support of 15 cmH_2_O. The patient was commenced on a morphine and midazolam infusion for pain and agitation. His Richmond analgesia and sedation score ranged between −2 (light sedation) and 0 (alert and calm). As part of the clinical assessment, respiratory ultrasound was performed which revealed an akinetic left hemidiaphragm, a mobile right hemidiaphragm and a clear lung parenchyma. A repeat ultrasound on the following day revealed an akinetic right hemidiaphragm but a mobile left hemidiaphragm (see Additional file 1 for video of the ultrasound images). All ultrasound images were taken during quiet breathing by the same physician (KH). A Vivid q GE Healthcare ultrasound machine (GE Healthcare, Wauwatosa, WI) with a curved array 4C-RS probe was used. The patient was examined in a supine position. The same landmarks and technique were used for both hemidiaphragms. The probe was placed laterally and perpendicularly on each lateral chest wall in a lower intercostal space between the mid and posterior axillary line. Therefore, the direction of the beam was perpendicular or near perpendicular to the craniocaudal axis during breathing. After a brief period of quiet consistent breathing, we obtained multiple video clips of each hemidiaphragm with a minimum of three successive respiratory cycles. We recorded at least three video clips that contained the maximum possible length of the hemidiaphragm. The images during forced breaths, sighs, and during suctioning were excluded [4].

CIDP was strongly suspected and the patient was empirically treated with intravenous immunoglobulin 0.4 g/kg for 5 days and prednisolone of 60 mg daily. Computerized tomography and magnetic resonant imaging of the brain were unremarkable. Magnetic resonant imaging of the spine revealed thickening and enhancement of the exiting spinal nerve roots and the cauda equina. Cerebrospinal fluid examination showed an elevated protein of 26.07 d/L (normal range 20.15–0.45), glucose 4.8 mmol/L (normal range 2.0–3.9), no red cells, white cell count 10 × 10^6^/L, mononuclear 9 × 10^6^/L and polymorphs 1 × 10^6^/L. There was no evidence of infection. A nerve conduction study revealed electrophysiological evidence of a polyneuropathy affecting motor fibers with mixed axonal and demyelinating features, with prolonged tibial and ulnar motor latencies and a reduction in motor conduction velocities.

Although the patient remained alert and cooperative, he was difficult to wean from mechanical ventilation. Tracheostomy was performed and he was gradually weaned off the mechanical ventilation. A repeated lung ultrasound (see Additional file 1) on day 10 showed mobility in both hemidiaphragms, though with reduced excursion (0.6 cm left and 0.8 cm right). The neurological abnormalities gradually resolved after a complete course of intravenous immunoglobulin.

The tracheostomy tube was successfully removed on day 22 from the date of admission to the intensive care unit. Computerized tomography of the chest abdomen and pelvis revealed extensive mediastinal, subcarinal, retrocrural, retroperitoneal and mesenteric lymphadenopathy as well as hepatomegaly and splenomegaly. A right retroperitoneal node biopsy confirmed diffused large B cell lymphoma. He was treated with R-CHOP chemotherapy (Rituximab, Cyclophosphamide, Doxorubicin, Vincristine and Prednisolone). The patient made a good recovery. He was discharged from the hospital to the care of the Haem-Oncology team as an outpatient.

## Conclusions

This case reports the unusual finding of alternating phrenic nerve palsy in CIDP manifested on bedside diaphragmatic ultrasound in real time. This phenomenon has not been described previously in CIDP. Our patient developed respiratory failure with normal chest radiography despite unilateral diaphragmatic paralysis, which would have been missed if ultrasound was not employed.

CIDP is a chronic acquired immune-mediated demyelinating polyneuropathy, which has been reported to be associated with non-Hodgkins lymphoma. The patient responded to immunotherapy and there was no evidence at the time or in subsequent follow-up to suggest that the patient had neurolymphomatosis. Respiratory failure develops secondary to phrenic neuropathy and denervation of respiratory muscles in demyelinating neuropathies [5]. It can be unilateral or bilateral [1, 6]. It is reversible when it is appropriately treated [2]. The clinical course of CIDP is characterized as relapsing or progressive [6].

Ultrasound is a non-invasive portable imaging technology that is available in most intensive care units. It is a bedside test with an ability to provide valuable information about respiratory sufficiency and diaphragmatic function in real time [3, 7]. In this case, ultrasound was a useful bedside tool that uncovered a new clinical observation pertinent to the neuroclinical course of CIDP.

## Additional file

10.1186/s13089-015-0033-5 Left and Right hemidiaphragm movements at day 1, 2 and 10.
